# Lactate clearance as a prognostic marker of mortality in severely ill febrile children in East Africa

**DOI:** 10.1186/s12916-018-1014-x

**Published:** 2018-03-09

**Authors:** A. Aramburo, Jim Todd, Elizabeth C. George, Sarah Kiguli, Peter Olupot-Olupot, Robert O. Opoka, Charles Engoru, Samuel O. Akech, Richard Nyeko, George Mtove, Diana M. Gibb, Abdel G. Babiker, Kathryn Maitland

**Affiliations:** 1 Royal Brompton & Harefield NHS Foundation Trust, Sydney Street, London, SW3 6NP UK; 2 London School of Hygiene and Tropical Medicine, 15-17, Tavistock Place WC1H 9SH, London, WC1H 9SH UK; 3 Medical Research Council Clinical Trials Unit (MRC CTU) at UCL, 125 Aviation House, Kingsway, London, WC2B 6NH UK; 4 Department of Paediatrics, Mulago Hospital, Makerere College of Health Sciences, PO Box 7072, Kampala, Uganda; 5 Department of Paediatrics, Mbale Regional Referral Hospital, Pallisa Road, PO Box 291, Mbale, Uganda; 6 Mbale Clinical Research Institute (MCRI), Plot 29-33 Pallisa Rd, PO Box 1966, Mbale, Uganda; 7 Department of Paediatrics, Soroti Regional Referral Hospital, PO Box 289, Soroti, Uganda; 8 Kilifi Clinical Trials Facility, KEMRI-Wellcome Trust Research Programme, PO Box 203, Nairobi, Kenya; 9 St Mary’s Hospital, Lacor, PO Box 180, Gulu, Uganda; 10 Teule Hospital, PO Box 81, Muheza, Tanzania; 11 Department of Paediatrics, Faculty of Medicine, Imperial College, W2 1PG, London, UK

**Keywords:** Hyperlactataemia, Children, Lactate clearance, East Africa, Mortality, Hospital admission, Clinical trials, Randomised, Sepsis, Malaria

## Abstract

**Background:**

Hyperlactataemia (HL) is a biomarker of disease severity that predicts mortality in patients with sepsis and malaria. Lactate clearance (LC) during resuscitation has been shown to be a prognostic factor of survival in critically ill adults, but little data exist for African children living in malaria-endemic areas.

**Methods:**

In a secondary data analysis of severely ill febrile children included in the Fluid Expansion as Supportive Therapy (FEAST) resuscitation trial, we assessed the association between lactate levels at admission and LC at 8 h with all-cause mortality at 72 h (d72). LC was defined as a relative lactate decline ≥ 40% and/or lactate normalisation (lactate < 2.5 mmol/L).

**Results:**

Of 3170 children in the FEAST trial, including 1719 children (57%) with *Plasmodium falciparum* malaria, 3008 (95%) had a baseline lactate measurement, 2127 (71%) had HL (lactate ≥ 2.5 mmol/L), and 1179 (39%) had severe HL (≥ 5 mmol/L). Within 72 h, 309 children (10.3%) died, of whom 284 (92%) had baseline HL. After adjustment for potential confounders, severe HL was strongly associated with mortality (Odds Ratio (OR) 6.96; 95% CI 3.52, 13.76, *p* < 0.001). This association was not modified by malaria status, despite children with malaria having a higher baseline lactate (median 4.6 mmol/L vs 3 mmol/L; *p* < 0.001) and a lower mortality rate (OR = 0.42; *p* < 0.001) compared to non-malarial cases. Sensitivity and specificity analysis identified a higher lactate on admission cut-off value predictive of d72 for children with malaria (5.2 mmol/L) than for those with other febrile illnesses (3.4 mmol/L).

At 8 h, 2748/3008 survivors (91%) had a lactate measured, 1906 (63%) of whom had HL on admission, of whom 1014 (53%) fulfilled pre-defined LC criteria. After adjustment for confounders, LC independently predicted survival after 8 h (OR 0.24; 95% CI 0.14, 0.42; *p* < 0.001). Absence of LC (< 10%) at 8 h was strongly associated with death at 72 h (OR 4.62; 95% CI 2.7, 8.0; *p* < 0.001).

**Conclusions:**

Independently of the underlying diagnosis, HL is a strong risk factor for death at 72 h in children admitted with severe febrile illnesses in Africa. Children able to clear lactate within 8 h had an improved chance of survival. These findings prompt the more widespread use of lactate and LC to identify children with severe disease and monitor response to treatment.

**Trial registration:**

ISRCTN69856593 Registered 21 January 2009.

**Electronic supplementary material:**

The online version of this article (10.1186/s12916-018-1014-x) contains supplementary material, which is available to authorized users.

## Background

Infection is a leading cause of death in children under 5 years of age in resource-poor countries in Africa, mainly related to pneumonia, diarrhea, and malaria [1]. Irrespective of the underlying aetiology, children with severe infection in these settings usually present critically ill to health units with basic facilities, with most deaths occurring within the first 24 h of admission [2]. Immediate recognition and prompt appropriate resuscitation of those children at highest risk of death are crucial to improve survival. Clinical criteria have been proposed by the World Health Organization (WHO) [3, 4], and practical clinical bedside risk scores have been evaluated to identify those at greatest risk of death [5]; however, data on point-of-care diagnostics to refine and target management at the point of triage are still lacking.

Hyperlactataemia (HL) is a well-known biomarker of severe disease that strongly predicts death in paediatric and adult patients with bacterial sepsis and malaria [6–9]. Its prognostic value, availability of point-of-care non-invasive testing, and immediate turnaround time have made lactate measurement one of the most widely recommended tools for early recognition and risk stratification of patients with severe sepsis [10–12]. In malaria-endemic areas, however, the use of point-of-care lactate measurement is controversial due to lack of data to support wider implementation [5, 13]. Nevertheless, therapeutic strategies aimed at decreasing lactataemia in serial determinations of lactate clearance (LC) could be a simple tool and may be clinically more valuable than initial single lactate measurements, helping to guide resuscitation and potentially being a cost-effective measure in these settings.

In recent years, several studies including two meta-analyses have demonstrated that early LC and lactate normalisation are powerful independent predictors of survival in critically ill adults [14–17]. Furthermore, two randomised clinical trials [18, 19] showed that targeting initial treatment to a pre-specified LC was associated with non-inferior or even superior outcomes compared to central venous oxygen saturation, a surrogate marker of cardiac output and a standard invasive therapeutic goal in sepsis. To date, however, only a few small studies have explored the prognostic value of LC in severely ill children with sepsis or malaria [20–22]. A large multicentre randomised control trial of fluid resuscitation strategies in severely ill febrile children in east Africa, the Fluid Expansion as Supportive Therapy (FEAST) trial, ISRCTN69856593 [23], provided a good opportunity to explore this question further.

The main aim of the current study was to assess the prognostic value of LC at 8 h on all-cause mortality at 72 h (d72) in a large cohort of severely ill febrile children in a malaria-endemic area. Secondary aims were to confirm the association between HL and d72, assess if this was modified by type of severe febrile illness (malaria vs non-malaria), and to determine the cut-off lactate level on admission that best predicts mortality in these children.

## Methods

The current study is a secondary analysis derived from the FEAST trial [23]. The overall aim of the trial was to answer whether rapid expansion of intravascular volume was safe and improved survival compared to no bolus (maintenance only). The study was conducted between January 2009 and January 2011 at six clinical centres, including large regional and district hospitals in three east African countries (Kilifi, Kenya; Muheza, Tanzania; and Kampala, Mbale, Soroti, and Lacor in Uganda). Malaria transmission in Kilifi and Muheza was predominantly seasonal and of moderate intensity during the period of the trial, whilst transmission at all sites in Uganda was perennial and intense.

### Study population

Children were eligible for the FEAST trial if they were between 60 days and 12 years of age, had a history of fever and/or abnormal temperature (pyrexia [≥ 37.5 °C] or hypothermia [< 36 °C]), and were admitted to hospital with severe illness (respiratory distress and/or impaired consciousness) plus at least one of the following signs of impaired peripheral perfusion: capillary refill time > 2 s; lower limb temperature gradient (defined as a notable temperature change from cold [dorsum of foot] to warm [knee] when running the back of the hand from the toe to the knee); weak radial pulse; or severe tachycardia (defined as heart rate > 180 beats per minute [bpm] for children < 1 year old, > 160 bpm for those 1 to 4 years old, > 140 bpm for those ≥ 5 years old). Children were excluded from the trial if they presented with severe acute malnutrition, gastroenteritis, burns, or surgical conditions. Children were randomised to receive immediate boluses of 20–40 mL/kg of 5% human albumin solution or 0.9% saline solution over 1 h (intervention group) following by maintenance fluids, or maintenance intravenous fluids only at 4 mL/kg/h (control group) until able to drink. The primary endpoint was mortality at 48 h after randomisation.

### Clinical care

Standardised case report forms were completed at enrolment and at specific time points during the first 48 h. At enrolment, lactate, haemoglobin, and glucose point-of-care measurements were conducted, together with an HIV antibody test, malaria blood film, and a rapid diagnostic test for malaria. At 8 h and 24 h, lactate and haemoglobin measurements were repeated. Lactate was measured using a Lactate Pro® (Arkray KDK, Kyoto, Japan) hand-held analyser, which was calibrated daily, and each time a new box of Lactate Pro® Test Strips was used. The test results, available in 60 s, displayed ‘LO’ if the lactate level was below 0.8 mmol/L and ‘HI’ if the lactate level was above 23.3 mmol/L. Out-of-range results were repeated for verification. Haemoglobin was determined with the HemoCue® Hb 301 system, Angelholm, Sweden. Children were managed on general paediatric wards, with no facilities for ventilation other than short-term ‘bag-and-mask’ support. Training in triage, identification, and definitions of adverse events related to fluid management (including transfusion) was given prior to and throughout the trial and was included in the trial manual of operations that was given to every team member. The main outcome of the FEAST trial [23] and the transfusion practices [24] used in the study have been reported in detail previously.

### Statistical analysis

The current secondary analysis was performed using the parent study database, cleaned and exported into STATA version 11 (StataCorp, LP, College Station, TX, USA). All children in the FEAST dataset with a valid lactate measurement at time of admission were included in the analysis, with a subsequent analysis of those who also had a lactate measurement 8 h after admission.

### Hyperlactataemia classification and lactate clearance definition

In the current study, we considered HL as a lactate > 2.5 mmol/L. We sub-categorised patients according to the lactate level on hospital admission as having moderate HL (lactate 2.5 to < 5 mmol/L), severe HL (lactate ≥ 5 mmol/L), or no HL (lactate < 2.5 mmol/L). An LC value was calculated for all children with an increased lactate level on admission (≥ 2.5 mmol/L) who were alive and had a lactate measurement at 8 h. Relative LC (percent) was defined by the equation [(lactate initial − lactate at 8 h)/lactate initial] × 100, for which lactate initial was the measurement at randomisation. Lactate normalisation was defined as a lactate decline to < 2.5 mmol/L at 8 h. The 8-h LC goal was defined by a relative lactate decrease of at least 40% from baseline and/or lactate normalisation.

Baseline clinical characteristics by survival status were described, combining randomisation groups. Frequencies and percentages were used for categorical variables, and medians with their inter-quartile range (IQR) for non-normally distributed continuous data. Baseline clinical characteristics were compared across outcome categories using the chi-squared test or the Wilcoxon signed-rank test as appropriate. Descriptive analysis reported both missing and available data.

The main exposure of interest was LC at 8 h. The primary outcome was all-cause in-hospital mortality at 72 h of randomisation (d72). As a first step in the LC analysis, the association between baseline HL and d72 was examined. For each of the exposures, HL and LC, the crude odds ratio (OR) with 95% confidence interval (CI) was calculated for the association with d72. To identify potential confounders, associations between patient characteristics and exposure and outcome variables were determined. Classic Mantel-Haenszel methods were used to explore for confounding and effect modification. A multivariable logistic regression model was fitted with a stepwise forward process selection to estimate the OR adjusted for several covariates simultaneously using all available data. Candidate variables were selected using previously defined risk factors of mortality and confounders identified in the univariate analysis. Age, sex, and study site were included *a priori* in both models due to their association with a wide range of health parameters. The effect of the trial intervention was included as a potential confounder and tested as an effect modifier of the association between LC and d72 with increased mortality in the FEAST trial. Likelihood ratio tests were used to assess for the model fit.

**Fig. 1:**
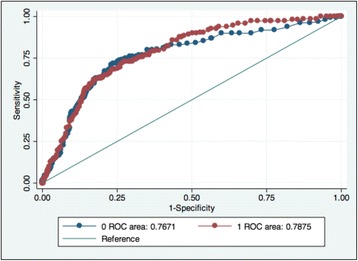
ROC curve for mortality at 72 h of randomisation based on lactate measurement at admission by malaria: 0 = no malaria and 1 = malaria

Receiver operating characteristic (ROC) curve analysis was used to determine the cut-off value for lactate level at hospital admission that would best predict death at 72 h. As children with malaria had a higher prevalence of severe HL despite a lower mortality rate, ROC curves were also built separately for children with and without malaria to assess if the best prognostic cut-offs differed (Fig. 1). A ROC curve was also fitted for d72 based on the regression model for LC at 8 h.

### Results

Of 3170 children randomised in the FEAST trial, 3008 (95%) had a lactate measurement on hospital admission and were included in the current analysis. In this group, there were 309 deaths (10.3%) in the first 72 h after randomisation, which accounted for 90% of the overall mortality by 28 days. The clinical and laboratory baseline characteristics of the children by primary outcome are summarised in Table 1. The median age was 24 months (IQR 13 to 39), and 1387 (46%) were female. The majority of children presented with respiratory distress (2463, 82%) and/or impaired consciousness (2326, 78%). All had at least one sign of impaired perfusion, including severe tachycardia (2120, 71%), a prolonged capillary refilling time of 2 s or longer (2030, 67%), or a lower limb temperature gradient (1777, 59%). Weak radial pulse volume, present in 635 children (21%) and bradycardia (in 36 children) were strongly associated with a poor outcome (unadjusted OR 4.24; 95% CI 3.30, 5.45; *p* < 0.001 and 13.13; 95% CI 6.62, 26; *p* < 0.001 respectively). Severe anaemia (Hb < 5 g/dL) was present in 958 children (33%) at admission. *Plasmodium falciparum* malaria was confirmed in 1719 children (57%) and HIV infection in 106 (4%) of the 2417 children tested. A blood culture was positive in 123 (12%) of the 1052 children in whom the test was performed.

**Table 1:** Clinical and laboratory characteristics on admission by in-hospital mortality at 72 h

	Survivors	Non-survivors	Total	Unadjusted OR of death (95% CI)	p value^a^
Number, N (%)^b^	2699 (90)	309 (10)	3008	–	–
General					
Female	1240/2699 (46)	147/309 (5)	1387/3008 (46)	1.07 (0.84, 1.35)	0.59
Age (months); median (IQR)^c^	24 (14, 39)	23 (11, 38)	24 (13, 38.5)	–	0.77^d^
Age < 12 months	494/2699 (16)	83/309 (3)	577/3008 (19)	1.64 (1.25, 2.15)	< 0.001
Middle-arm circumference ≤ 11.5 cm	56/2644 (1.95)	13/199 (0.5)	69/2843 (2.4)	3.23 (1.73, 6)	< 0.001
Axillary temperature > 39 °C	658/2694 (22)	45/309 (1)	703/3003 (23)	0.62 (0.44, 0.86)	< 0.01
Hypothermia (temperature < 36 °C)	133/2694 (4)	53/309 (2)	186/3003 (6)	3.99 (2.82, 5.64)	< 0.001
Respiratory					
Respiratory rate; median (IQR)^c^	57 (48, 67)	60 (50, 70)	57 (48, 67)	–	0.03^d^
Respiratory distress	2183/2688 (73)	280/308 (9)	2463/2996 (82)	2.31 (1.55, 3.46)	< 0.001
Deep breathing	1674/2697 (56)	261/308 (8)	1935/3005 (64)	2.47 (1.95, 3.14)	< 0.001
Hypoxaemia (oxygen saturation ≤ 90%)	521/2626 (18)	113/271 (4)	634/2897 (22)	2.89 (2.22, 3.76)	< 0.001
Crackles on auscultation	543/2697 (18)	112 /308 (4)	655/3005 (22)	2.27 (1.76, 2.92)	< 0.001
Cardiovascular					
Severe tachycardia^e^	1955 /2697(73)	165/308 (54)	2120/3005 (71)	0.44 (0.34, 0.56)	< 0.001
Bradycardia	15/2698 (0.6)	2/3071 (7)	36/3005 (1.2)	13.13 (6.62, 26)	< 0.001
Capillary refill time:					
< 2 s	921/2696 (34)	53/308 (17)	974/3004 (32)	1.00 (Ref.)^f^	
2–3 s	1130/2696 (42)	109/308 (35)	1239/3004 (41)	2.50 (1.83, 3.40)	< 0.001
≥ 3 s	645/2696 (24)	146/308 (47)	791/3004 (26)	2.87 (2.25, 3.66)	< 0.001
Lower limb temperature gradient^g^	1544/2699 (57)	233/309 (75)	1777/3008 (59)	2.29 (1.75, 3.01)	< 0.001
Weak radial pulse	486/2699 (18)	149/309 (48)	635/3008 (21)	4.24 (3.30, 5.45)	< 0.001
Moderate hypotension^h^	162/2664 (6)	40/289 (14)	202/2953 (7)	2.48 (1.71, 3.60)	< 0.001
Dehydration (sunken eyes or reduced skin turgor)	178/2697 (7)	51/307 (17)	229/3004 (8)	2.82 (2.01, 3.96)	< 0.001
Neurological					
Level of consciousness:					
Alert	657 /2696(24)	22/309 (7)	679/3005 (23)	1.00 (Ref.)^f^	
Prostrate^i^	1714/2696 (64)	171/309 (55)	1885/3005 (63)	2.98 (1.89, 4.69)	< 0.001
Coma^j^	325/2696 (12)	116/309 (38)	441/3005 (15)	10.66 (6.63, 17.14)	< 0.001
Convulsions in this illness	301/2698 (11)	48/309 (16)	349/3007 (12)	1.40 (1.00, 1.95)	0.05
Clinical anaemia					
Severe pallor^k^	969/2694 (36)	170 /309(55)	1139/3003 (38)	2.18 (1.71, 2.77)	< 0.001
Haemoglobinuria (history of dark urine)	339/2694 (1.4)	40/307 (13)	379/3001 (13)	1.21 (0.95, 1.54)	0.13
Jaundice	833/2695 (31)	133/309 (43)	966/3004 (32)	1.65 (1.30, 2.10)	< 0.001
Laboratory tests					
Lactate (mmol/L); median (IQR)^c^	3.4 (2.2, 6.9)	10.9 (5.2, 13.6)	3.8 (2.3, 8)	–	< 0.001^d^
Hyperlactataemia (lactate, mmol/L)					
No (< 2.5)	856/2699 (32)	25/309 (8)	881/3008 (29)	1.00 (Ref.)^f^	
Moderate (2.5–5)	902/2699 (33)	46/309 (15)	948/3008 (32)	1.75 (1.06, 2.87)	0.03
Severe (≥ 5)	941/2699 (35)	238/309 (77)	1179/3008 (39)	8.66 (5.6, 13.4)	< 0.001
Severe acidaemia:					
pH < 7.2	138/1816 (8)	70/208 (34)	208/2024 (10)	6.17 (4.35, 8.74)	< 0.001
Base deficit > −8 mmol/L	869/1809 (48)	170/203 (84)	1039/2012 (58)	5.57 (3.76, 8.26)	< 0.001
Bicarbonate < 15 mmol/L	588/1808 (3)	151/206 (73)	739/2014(37)	5.69 (4.07, 7.97)	< 0.001
Haemoglobin (g/dL):			2930		
≥ 10	648/2628 (25)	42/302 (14)	690/2930 (24)	1.00 (Ref.)^f^	
7 to < 10	720/2628 (27)	80/302 (27)	800/2930 (27)	1.71 (1.16, 2.53)	< 0.01
5 to < 7	437/2628 (17)	45/302 (15)	482/2930 (16)	1.59 (1.02, 2.46)	0.04
< 5	823 /2628(31)	135/302 (45)	958/2930 (33)	2.53 (1.76, 3.64)	< 0.001
Glucose (mmol/L); median (IQR)^c^	6.9 (5.5, 8.70)	5.95 (3.6, 9.1)	6.9 (5.4, 8.7)	–	< 0.001^d^
Hypoglycaemia (glucose < 2.5 mmol/L)	82/2615 (3)	48 /300(16)	130/915 (4)	5.88 (4.00, 8.66)	< 0.001
Blood urea nitrogen (BUN) ≥ 7.14 mmol/L	337/1819 (19)	90/205 (44)	427/2024 (21)	3.44 (2.54, 4.67)	< 0.001
Hyperkalaemia (≥ 6 mmol/L)	50/1768 (3)	37 /197(19)	87/1965 (4)	7.90 (4.98, 12.7)	< 0.001
Microbiology					
Malaria parasitaemia, positive^l^	1571/2695 (58)	148/307 (48)	1719/3002 (57)	0.67 (0.53, 0.84)	< 0.001
HIV antibody, positive	86/2197 (4)	20/220 (9)	106/2417(4)	2.46 (1.48, 4.08)	< 0.001
Blood culture, positive	106/947 (11)	17/105 (16)	123 /1052 (12)	1.53 (0.88, 2.68)	0.13

^a^Chi-squared test *p* values

^b^Of 3170 children enrolled in the FEAST trial, 162 did not have lactate measured at baseline

^c^IQR: percentile 25, percentile 75

^d^Wilcoxon rank sum test *p* value

^e^Severe tachycardia was defined as > 180 beats per minute (bpm) in children < 12 months, > 160 bpm in children aged 12 months to 5 years, and > 140 bpm for those aged > 5 years

^f^Ref: Reference group

^g^Temperature gradient was assessed by running the back of the hand from the toe to the knee; a positive temperature gradient was defined as a notable temperature change from cold (dorsum of foot) to warm (knee)

^h^Moderate hypotension was defined as systolic blood pressure of 50–75 mmHg in children younger than 12 months, 60–75 mmHg in those 12 months to years, and 70–85 mmHg in those older than 5 years, as measured by automated blood pressure monitor

^i^Prostration was defined as the inability of a child older than 8 months of age to sit upright or the inability of a child 8 months of age or younger to breast feed

^j^Coma was defined as the inability to localise a painful stimulus

^k^Pallor manifested in lips, gums, or inner eyelids

^l^Positive on quality-controlled malaria slide, or, if missing, on rapid diagnostic test

### Baseline lactate level and mortality

At hospital admission 2127 children (71%) had a raised baseline lactate (≥ 2.5 mmol/L). Of these, 948 children (32%) had moderate HL (lactate 2.5 to < 5 mmol/L) and 1179 (39%) had severe HL (lactate ≥ 5 mmol/L). Children with severe HL had a higher prevalence of other clinical and laboratory markers of disease severity, including hypoxaemia (*S*_p_O_2_ < 90% by pulse oximetry) (*p* < 0.001), coma (*p* < 0.001), severe anaemia (*p* < 0.001), or severe acidosis (*p* < 0.001). Children with malaria parasitaemia had higher mean baseline lactate (4.6 vs 3 mmol/L, *p* < 0.001) and prevalence of severe HL (46% vs 30%, *p* < 0.001) than those with other febrile illnesses.

Of the 309 children who died in the first 72 h, 238 (77%) had severe HL on admission and 46 (15%) had moderate HL. The median admission lactate in children who died was significantly higher (10.9 mmol/L; IQR 5.2–13.6) than in those who survived the first 72 h (3.4 mmol/L; IQR 2.2–6.9) (*p* < 0.001). Amongst those children who died, the median admission lactate was higher when death occurred in the first 8 h (12.2 mmol/L; IQR 8.6–13.9) than when it occurred between 8 and 72 h (7.65 mmol/L; IQR 4.1–12.6) or after 72 h (3.2 mmol/L; IQR 2.1–5.2) of the admission respectively.

In the univariate analysis, baseline severe HL strongly increased the odds of death at 72 h (OR 8.66; 95% CI 5.7, 13.2), whilst moderate HL had a weaker effect on mortality (OR 1.75; 95% CI 1.06, 2.87). This association did not significantly vary amongst children with and without malaria, indicating that severe HL was equally prognostic for both conditions. Evidence for an effect modification, however, was found between HL and hyperglycaemia (glucose ≥ 8.3 mmol/L) (heterogeneity test *p* < 0.01), elevated blood urea nitrogen (BUN) (heterogeneity test *p* < 0.001), and presence of crackles on auscultation (heterogeneity test *p* = 0.025) (Additional file 1: Supplementary tables).

After full adjustment for confounders (Table 2), severe HL on hospital admission remained strongly associated with death at 72 h (OR 6.96; 95% CI 3.52, 13.76), whilst moderate HL showed a weaker association (OR 1.57; 95% CI 0.80, 3.08).

**Table 2:** Multivariate analysis (logistic regression model) of clinical and laboratory factors at admission associated with mortality at 72 h of randomisation

	Categories	Adjusted OR of death (95% CI)	*p* value^a^
Age (months)	≥ 12	1.00 (Ref.)^b^	< 0.01
	< 12	1.98 (1.22, 3.2)	
Gender	Male	1.00 (Ref.)^b^	0.96
	Female	1.01 (0.71, 1.46)	
Site	Mbale	1.00 (Ref.)^b^	
	Kilifi	1.56(0.76, 3.23)	0.23
	Mulago	1.47 (0.80, 2.70)	0.21
	Soroti	1.16 (0.64, 2.14)	0.63
	Lacor	–	–
	Teule	2.04 (0.87, 4.76)	0.10
Lactate stratum (mmol/L)	No (< 2.5)	1.00 (Ref.)^b^	
	Moderate (2.5–5)	1.28 (0.62, 2.63)	0.50
	Severe (≥ 5)	6.96 (3.52, 13.76)	< 0.001
Level of consciousness	Alert	1.00 (Ref.)^b^	
	Prostration	1.41 (0.72, 2.76)	0.31
	Coma	6.87 (3.36, 14.04)	< 0.001
BUN (mmol/L)^d^	< 7.14	1.00 (Ref.)^b^	< 0.001
	≥ 7.14	2.92 (1.84, 4.62)	
Malaria	No	1.00 (Ref.)^b^	< 0.001
	Yes	0.45 (0.29, 0.69)	
Severe anaemia (Hb < 5 g/dL)	No	1.00 (Ref.)^b^	0.37
	Yes	0.81 (0.50, 1.29)	
Hyperglycaemia^c^ (glucose ≥ 8.3 mmol/L)	No	1.00 (Ref.)^2^	0.55
	Yes	0.84 (0.55, 1.30)	
HIV	No	1.00 (Ref.)^2^	0.05
	Yes	2.16 (1.01, 4.60)	

^a^Wald test *p* values

^b^Ref: Reference group

^c^Moderate evidence of interaction, stratum-specific odds ratios are shown in Additional file 1

^d^Blood Urea Nitrogen

Coma was a strong independent risk factor (OR 6.87; 95% CI 3.36, 14.04), and elevated BUN and HIV infection were moderately strong independent risk factors of death identified in the model. Whilst a positive malaria test appeared to be more strongly associated with higher likelihood of survival, this cannot be interpreted as a protective effect of parasitaemia per se, since the model was controlling for other factors which are likely impacted by malaria and strongly associated with mortality.

The multivariable regression model accounting for the interaction between HL and hyperglycaemia demonstrated a weakened association between HL and mortality in the presence of hyperglycaemia (OR 3.22; 95% CI 1.56, 6.67; *p* < 0.01), although the effect of HL on the risk of mortality in the absence of hyperglycaemia (OR 8.55; 95% CI 4.63, 15.78; *p* < 0.001) was slightly greater. Furthermore, logistic regression analysis accounting for the additional interactions identified demonstrated that the association of HL with d72 was considerably lower in the 427 children (21%) with high admitting BUN (OR 2.33; 95% CI 1.11, 4.89; *p* = 0.025) and much higher in the 655 children (22%) with crackles on auscultation (OR 29.45; 95% CI 10.13, 85.55; *p* < 0.001) (Additional file 1).

### Lactate clearance at 8 h and mortality

Of all children included in the analysis, 2748/3008 (91.4%) were alive and had a blood lactate level measured at 8 h of randomisation; 1906 (63.3%) of these had an elevated lactate level (≥ 2.5 mmol/L) on admission and contributed to the LC analysis. Table 3 summarises the absolute and relative LC and lactate normalisation parameters at 8 h by primary outcome. Overall, the median lactate at 8 h was 3.4 mmol/L (IQR 2.3–5.1), the median absolute LC (change from baseline in lactate level) was 1.6 mmol/L (IQR 0.1–4.6), and the median relative LC (from baseline value) was 36% (IQR 3.3–59%). Children who survived to 72 h had a lower median lactate (3.3 vs 5.4 mmol/L, *p* < 0.001) and a higher relative LC (37% vs 17%, *p* < 0.01) at 8 h. Median relative LC was significantly higher in children with severe HL (54%; IQR 28–70) than in those with moderate HL (15%; IQR −19 to 38) (*p* < 0.001). Children with malaria had a similar relative LC as those with other severe febrile illnesses (37% vs 35%, *p* = 0.4).

**Table 3:** Lactate clearances within first 8 h of randomisation in survivors and non-survivors at 72 h

	Survivors	Non-survivors	Total	Unadjusted OR of death (95% CI)	p value^a^
Lactate clearance at 8 h amongst children with an abnormal lactate on admission ≥ 2.5 mmol/L^b^					
Number, N (%)	1792 (94)	114 (6)	1906	–	–
Lactate at 8 h (mmol/L); median (IQR)^c^	3.3 (2.3, 5)	5.4 (3.4, 10)	3.4 (2.3, 5.1)	–	< 0.001^d^
Absolute LC (mmol/L); median (IQR)^c^	1.7 (0.2, 4.5)	1.3 (−0.5, 4.7)	1.6 (0.1, 4.6)	–	0.12^d^
Relative LC (%); median (IQR)^c^	37 (3.7, 59.5)	17 (−6, 51)	36 (3.33, 59)	–	0.003^d^
Relative LC < 10%	516/1792 (29)	50/114 (44)	566/1906 (30)	1.93 (1.31, 2.84)	< 0.001
Relative LC ≥ 40%	840/1792 (47)	38/114 (33)	878/1906 (46)	0.57 (0.38, 0.85)	< 0.01
Lactate normalisation^e^	540/1792 (30)	14/114 (12)	554/1906 (29)	0.33 (0.18, 0.57)	< 0.001
Relative LC ≥ 40% and/or lactate normalisation	973/1792 (54)	41/114 (36)	1014/1906 (53)	0.47 (0.32, 0.70)	< 0.001
Lactate clearance at 8 h amongst those with lactate on admission ≥ 5 mmol/L^f^					
N (%)	919 (92)	85 (8)	1004	–	–
Lactate at 8 h; median (IQR)^c^	3.9 (2.7, 6)	6.8 (4.1, 11.3)	4.1 (2.8, 6.3)	–	< 0.001^d^
Absolute LC (mmol/L); median (IQR)^c^	4.4 (2.4, 7.7)	2.3 (0, 5.8)	4.3 (2.3, 7.6)	–	< 0.001^d^
Relative LC (%); median (IQR)^c^	55 (32, 71)	23 (0, 62)	54 (28, 70)	–	< 0.001^d^
Relative LC < 10%	116 /919(13)	33/85 (39)	149/1004 (15)	4.40 (2.70, 7.16)	< 0.001
Relative LC ≥ 40%	628/919 (68)	33/85 (39)	661/1004 (66)	0.29 (0.18, 0.47)	< 0.001
Lactate normalisation^e^	200/919 (22)	6/85 (7)	206/1004 (21)	0.27 (0.12, 0.64)	0.001
Relative LC ≥ 40% and/or lactate normalisation	628/919 (68)	33/85 (39)	661/1004 (66)	0.29 (0.18, 0.47)	< 0.001

^a^Chi-squared test *p* values

^b^Total of 811 children with normal lactate levels on admission (< 2.5 mmol/L) excluded

^c^Ref: Reference group

^d^Wilcoxon rank-sum test *p* values

^e^Lactate decline < 2.5 mmol/L in the first 8 h after randomisation

^f^1713 children with lactate levels on admission < 5 mmol/L excluded

At 8 h, 566 children (30%) had a relative LC < 10%, whereas a total of 1014 (53%) met the pre-defined LC criteria (LC decline ≥ 40% [878, 46%] and/or lactate normalisation [< 2.5 mmol/L] [554, 29%]). After full adjustment for confounders, including the effects of lactate level on admission and the FEAST trial (fluid) intervention arm, failure to clear lactate (LC < 10%) at 8 h of treatment was strongly associated with death at 72 h (OR 4.62; 95% CI 2.7, 8; *p* < 0.001). Furthermore, achieving the combined LC pre-defined goal was the strongest prognostic factor of survival (OR 0.24; 95% CI 0.14, 0.42; *p* < 0.001) compared to relative LC or lactate normalisation alone (Table 4).

**Table 4:** Multivariable analysis of clinical and laboratory factors associated with death at 72 h including LC at 8 h post-randomisation

	Categories	Adjusted OR of death (95% CI)	p value^a^
LC criteria^b^ at 8 h	No	1.00 (Ref.)^c^	< 0.001
	Yes	0.24 (0.14, 0.42)	
Hyperlactataemia on admission (≥ 5 mmol/L)	No	1.00 (Ref.)^c^	< 0.001
	Yes	6.61 (3.56, 12.30)	
FEAST fluid intervention arm	Control	1.00 (Ref.)^c^	< 0.01
	Fluid bolus	2.48 (1.36, 4.51)	
Malaria	No	1.00 (Ref.)^c^	0.01
	Yes	0.52 (0.31, 0.87)	
Severe anaemia	No	1.00 (Ref.)^c^	0.05
	Yes	0.57 (0.32, 0.99)	
Level of consciousness	Alert	1.00 (Ref.)^c^	
	Prostration	0.87 (0.38, 2)	0.78
	Coma	4.29 (1.8, 10.24)	0.001
BUN (mmol/L)	< 7.14	1.00 (Ref.)^c^	< 0.001
	≥ 7.14	3.13 (1.80, 5.43)	
Age (months)	≥ 12	1.00 (Ref.)^c^	< 0.001
	< 12	3.37 (1.95, 5.82)	
Gender	Male	1.00 (Ref.)^c^	0.66
	Female	1.11 (0.69, 1.81)	
Site	Mbale	1.00 (Ref.)^c^	
	Kilifi	2.16 (0.82, 5.75)	0.12
	Mulago	2.73 (1.29, 5.78)	< 0.01
	Soroti	1.31 (0.58, 2.95)	0.52
	Lacor	3.12 (1.19, 8.2)	0.02
	Teule	2.93 (1.05, 8.22)	0.04

^a^Wald test *p* values

^b^LC defined as a relative lactate decline ≥ 40% and/or lactate normalisation (lactate < 2.5 mmol/L) at 8 h of randomisation

^c^Reference group

Persistent severe HL at 8 h remained a strong independent risk factor of death at 72 h (OR 4.60; 95% CI 2.61–8.1; *p* < 0.001). Of note, as previously demonstrated in the FEAST trial, fluid boluses were strongly associated with death at 72 h (OR 2.48; 95% CI 1.36, 4.51; *p* < 0.01) in the full-adjusted logistic regression model.

### ROC analysis and lactate cut-offs

**Fig. 2:**
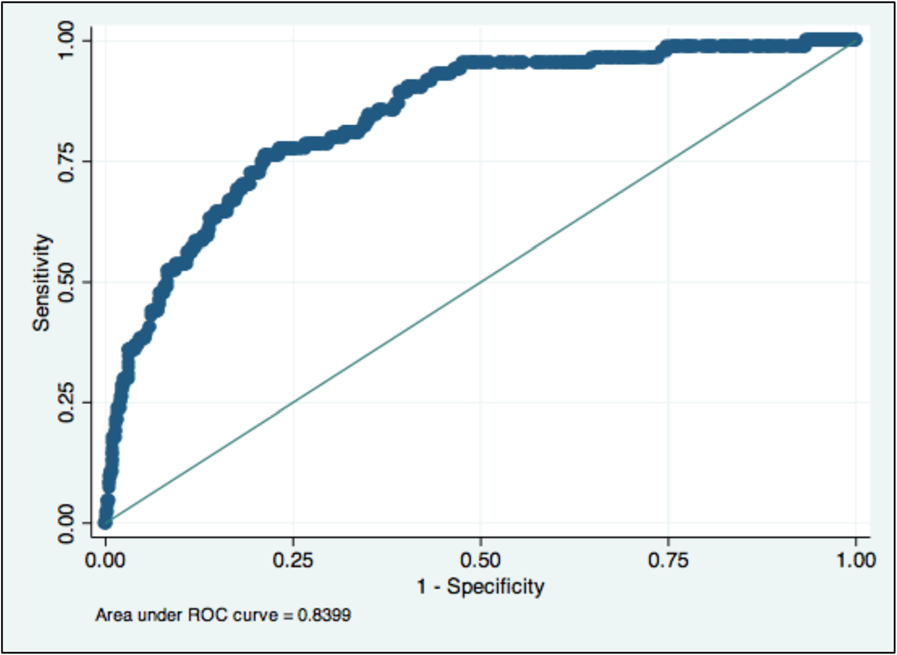
ROC curve for mortality at 72 h of randomisation based on logistic regression model for lactate clearance (LC) at 8 h. LC was defined as a relative LC ≥ 40% and/or lactate normalisation (lactate < 2.5 mmol/L) within the first 8 h of treatment

The area under the receiver operator curve (AUROC) for d72 based on lactate at admission was 0.77 (95% CI 0.74–0.80), with no statistically significant difference in AUROC amongst children with and without malaria (*p* = 0.47) (Fig. 1). The AUROC based on lactate at 8 h was 0.73 (95% CI 0.68–0.77), with again no difference by malaria status (*p* = 0.55). The overall AUROC based on LC at 8 h was 0.58 (95% CI 0.53–0.64), and for the group of children with severe HL (> 5 mmol/L) on admission it was greater (0.68; 95% CI 0.62–0.75). Lastly, the AUROC for d72 based on a logistic regression model for LC at 8 h was even more predictive as 0.84 (95% CI 0.80–0.88) (Fig. 2).

Sensitivity and specificity analysis identified that for children without falciparum malaria a lactate level on admission of ≥ 3.4 mmol/L had a sensitivity to predict mortality at 72 h of 80% and a specificity of 61.6%, whereas for children with malaria a sensitivity of 80% corresponded with a lactate cut-off value of 5.2 mmol/L (specificity of 59.2%). For a baseline lactate level of ≥ 5 mmol/L, the sensitivity in children without malaria was 71.7% (specificity 75.53%), whereas in those with malaria it was 82.43% (specificity 57.7%). This indicates that in non-malaria cases, lower levels of HL have a similar effect on 72-h mortality.

## Discussion

Our study confirms the association between HL on hospital admission and mortality, and it demonstrates that a failure to clear lactate (relative LC < 10%) within the first 8 h of treatment is a relevant prognostic factor of early death in severely ill febrile children in a malaria-endemic area irrespective of the underlying diagnosis (malaria, sepsis, or other severe febrile illness). Importantly, this is the first paediatric study to demonstrate that a relative LC ≥ 40% and/or lactate normalisation within the first 8 h of treatment strongly predicts 72-h survival in this context.

These results, derived from a very large multicentre randomised trial, are compelling and raise the question of whether LC could be a simple, valid and cost-effective risk-stratification tool, as well as a potential therapeutic target to guide resuscitation in children with severe febrile illnesses in resource-poor settings. Furthermore, it is not limited to children with specific diagnoses, but rather covers different presentation syndromes, which reflects the population of children presenting to hospital in the setting in which the trial was conducted. Lastly, for research studies the inclusion of lactate level could be used for inter-site or inter-centre comparisons of disease severity.

Despite a paucity of paediatric data, our findings are supported by the literature. A recent meta-analysis [25] and a large systematic review [17] of acutely ill adults have reported a consistent lower mortality associated with LC, not limited to septic patients and regardless of the initial lactate value. Also, a recent secondary analysis of two large clinical trials on antimalarials has shown that lack of LC is an independent predictor of death in adults with severe malaria [26]. Amongst the few paediatric studies published, Krishna and colleagues measured serial lactate concentrations during 24 h in a small cohort of 115 children with severe malaria in the Gambia, concluding that sustained HL was the most powerful prognostic indicator of fatal outcome [21]. In another cohort of 65 children admitted to a paediatric intensive care unit (PICU) with septic shock in South Korea, Kim *et al.* [22] found that the area under the curve (AUC) of serial lactates measured during the first 24 h had a strong predictive power of mortality at 28 days (ROC AUC 0.828). Munde *et al*. [27] recently reported that a relative LC < 30% at 6 h predicted mortality in PICU children in India comparably to the more complex Paediatric Risk of Mortality (PRISM) score, standard in paediatric intensive care.

It is therefore possible that failure to clear lactate within the first hours of therapy could serve as a simpler risk stratification tool in malaria-endemic areas, potentially more reliable than absolute lactate values alone and easier to apply than more complex clinical scores. However, to have an outcome benefit, a similar lack of LC effect should likely be shown at an interval shorter than 8 h that would allow a therapeutic intervention or the appropriate recruitment into clinical trials of those children with a higher chance to die.

Moreover, to demonstrate an association between LC and survival in severely ill febrile children in a malaria-endemic area is relevant at least in two aspects. On one hand, LC could serve as a valid surrogate endpoint of clinical trials of malaria aiming to improve mortality, as recently demonstrated by Jeeyapant *et al.* in a large cohort of adult patients with malaria [26]. Thus, one could hypothesise that LC might help to guide the initial resuscitation of these children in a resource-poor setting, where invasive monitoring and intensive care is mostly unavailable. To date two multicentre randomised trials have assessed the clinical value of resuscitation strategies that included LC as a target in adults admitted to intensive care units, showing that quantitative resuscitation based on LC was non-inferior or even superior to that based on *S*_cv_O_2_ alone [18, 19], and the most recent Surviving Sepsis Campaign guidelines suggest targeting resuscitation to lactate normalisation in adult septic patients with HL, although accept a weak evidence exists for this recommendation [28].

Unfortunately, the observational design of our study does not allow one to answer whether targeting resuscitation to achieve a specific relative LC or lactate normalisation improves outcomes in severely ill febrile children in this context. Interestingly, a recent study including 218 adult patients with severe infection at a regional referral hospital in Uganda [29] questioned whether serial assessment of vital signs combined with point-of-care lactate at 6 h could be a feasible method of monitoring patients being resuscitated from severe sepsis or malaria in a resource-poor setting. Despite an improvement in vital signs and lactate values at 6 h of resuscitation, the authors could not demonstrate an association between an LC ≥ 10% and improved in-hospital mortality. This study, however, was observational with a limited sample size where more than half of the participants had advanced HIV infection, it did not use a pre-defined treatment algorithm to target LC, and it did not report adjusted OR for the association of interest.

Some other limitations of our study should be taken into account. First, despite the optimal LC threshold or the LC rate not having been clearly defined, many studies on LC in sepsis have used a relative clearance rate of 10–20% in repeated samples measured at 2- to 3-h intervals during the first 6 h of resuscitation. Our definition of LC criteria was based on published data [14, 18, 19], the median LC value in our cohort, and the 8-h interval between lactate samples. A more extreme or loose definition could have yielded slightly different results. Moreover, the fact that LC in the FEAST trial was calculated at 8 h after randomisation for all participants prevents one from determining the precise moment when lactate normalisation or LC occurred for each individual. If some of the participants cleared their lactate significantly earlier than 8 h and that was associated with a better outcome, the true effect of lactate normalisation/LC on survival could be underestimated. However, 169/341 children (49.6%) died before 8 h, which again could have resulted in a relevant underestimation of the study results. The key point is that we chose the 8-h correction as this could be compared to other studies [26–28]; given the high proportion of mortality that occurred before this time point, an early assessment of LC may be more relevant to this setting to identify high-risk patient and initial resuscitation (either at 2 or 4 h). Lastly, LC can be potentially useful when baseline HL exists, which was not the case for one third of our participants.

Whilst lactate levels can falsely increase with haemolysis, frequent with difficult phlebotomies or with long time span from extraction to processing, blood samples in the FEAST trial were obtained from a free-flowing vein and immediately measured at the bedside. Arterial lactates are recommended over peripheral venous lactates in sepsis studies looking at absolute values and clearance rate, due to poor agreement between both parameters [30]. Nevertheless, there are technical and ethical challenges in obtaining arterial samples in un-sedated children, and the strong prognostic correlation with high admission venous lactate and LC with survival outcomes suggests that pragmatically venous samples should be suitable for critically sick children in resource-limited hospitals. Lastly, lactate levels may increase with hyperglycaemia, unrelated to a tissue oxygen debt but related to a stress-induced increased glucose turnover. This so-called type B lactic acidosis usually resolves quickly after normalisation of glycaemia [31]. Only 6% of participants in the analysis had hyperglycaemia on admission, so it is unlikely this would have introduced a bias in the overall analysis. However, some evidence of an effect modification was found between HL and hyperglycaemia (see Additional file 1), with a weakened association between HL and death in the presence of hyperglycaemia.

Relevant to the FEAST Paediatric Emergency Triage (PET) clinical score [5], which includes the presence of crackles (lung crepitations) on auscultation as a predictive maker of death, we too found that lung crepitations in association with high lactate strongly predicts 72-h mortality (OR 29.90; 95% CI 11.43–78.14) in children with shock, which most likely indicates the aetiology was sepsis due to pneumonia. This observation may help refine definitions of severe pneumonia, which are broad and non-specific. Interestingly, in the PET score analysis hypoxaemia (pulse oximetry reading < 90%) did not independently predict poor outcome [5]. In this analysis, hypoxaemia was found to be independently associated with death at 72 h (OR 1.72, *p* = 0.01); however, as it did not confound or modify the association between HL and d72, it was not included in the logistic regression model. Moreover, children septic with pneumonia (defined as crackles on auscultation and presence of hyperlactataemia) were more likely to suffer from hypoxaemia (OR = 2.58; *p* < 0.0001). Owing to the poor specificity of signs for pneumonia, WHO promotes the use of pulse oximeters to direct children with signs of pneumonia for oxygen therapy. Yet, pulse oximetry remains poorly implemented [32]. One example, therefore, of the utility of the PET score and/or use of lactate screening at admission is using lung crepitations in the future to screen for clinical trials examining novel therapies specifically for pneumonia in those at high risk for poor outcome.

Lastly, our findings suggest that the predictive values of lactate on admission for mortality are lower in children without malaria (3.4 mmol/L) than for those with malaria (5.2 mmol/L). This is important, as a detailed pathogenesis of HL in septic shock and and in malaria is still imperfectly understood and may actually differ. Whereas in sepsis HL is mostly viewed as a result of anaerobic metabolism secondary to systemic hypoperfusion [33], HL in malaria is likely an even more complex phenomenon, involving additional factors like sequestration of parasitised erythrocytes in the microcirculation, acute severe haemolytic anaemia, seizure activity, and end-products of parasite metabolism [34–36]. We have previously described the relationship between baselines haemoglobin and lactate (Additional file 2). This likely diverse pathogenesis of HL in bacterial sepsis and malaria could have implied a different prognostic value for mortality that would explain the different definitions of HL used in studies of sepsis and malaria. Thus, while the current consensus cut-off value for sepsis is a lactate > 2 mmol/L [37], most studies on severe malaria, where HL is particularly frequent and profound, use a lactate cut-off of > 5 mmol/L, following the WHO definition criteria [38]. Our study indicates that appropriate lactate cut-off values, lower for children without malaria, should be included in the design of therapeutic algorithms for early risk stratification of severely ill children admitted to hospital in malaria-endemic areas.

## Conclusions

In conclusion, severe HL, defined as a venous lactate level ≥ 5 mmol/L on hospital admission, is a strong risk factor for death within the first 72 h in children with severe febrile illnesses in east Africa, independently of the underlying diagnosis. Failure to clear lactate (relative LC ≤ 10%) within 8 h is also strongly prognostic of death, which could serve as a simple risk-stratification tool or a surrogate endpoint of mortality in clinical trials. Those children able to normalise or clear their lactate by at least 40% within 8 h have an improved chance of survival. An LC measured at an earlier time point may have yielded even more relevant results owing to the high mortality prior to 8 h that occurred in our cohort. Whether LC alone, or in combination with other clinical markers, can be used as a cost-effective therapeutic target to guide initial resuscitation and ultimately improve clinical outcomes of severely ill febrile children in malaria-endemic areas, where lack of basic patient monitoring and intensive therapies is standard, remains uncertain. Given severe infection is the most common cause of death in children under 5 years of age in these settings, and that early and aggressive initial resuscitation is key for survival, this question warrants further investigation. These data provide the rationale for a clinical trial of lactate clearance as an early therapeutic resuscitation goal in children with severe infection in malaria-endemic areas. We encourage the use of point-of-care lactate testing whenever possible in limited-resource settings to identify high-risk patients.

## Additional files

Additional file 1: Multivariable analysis (logistic regression model) of clinical and laboratory factors associated with death at 72 h: fully adjusted OR (including interaction parameter between HL and hyperglycaemia, blood urea nitrogen levels, and crackles on auscultation). (DOCX 106 kb)

Additional file 2: Plots of the relationship between haemoglobin and lactate and mortality estimated from the adjusted Cox regression model. Originally published in BMC Medicine 13:174 by George et al. [5]. (PNG 80 kb)

## Abbreviations

d72: Mortality at 72 h of hospital admission
FEAST: Fluid Expansion as Supportive Therapy
Hb: Haemoglobin
HL: Hyperlactataemia
LC: Lactate clearance
