# Supplementary material for: Lactate clearance as a prognostic marker of mortality in severely ill febrile children in East Africa
Source: BMC Med. 2018 Mar 9;16:37. doi: 10.1186/s12916-018-1014-x (PMC5844084; doi:10.1186/s12916-018-1014-x)
Supplement: Supplementary file 1 — Multivariable analysis (logistic regression model) of clinical and laboratory factors associated with death at 72 h: fully adjusted OR (including interaction parameter between HL and hyperglycaemia, blood urea nitrogen levels, and crackles on auscultation). (DOCX 106 kb) [file 12916_2018_1014_MOESM1_ESM.docx]

| **Multivariable analysis (logistic regression model) of clinical and laboratory factors on admission associated with death at 72 hours: fully adjusted OR (including interaction parameter between HL and hyperglycaemia)** | | | | | |
| --- | --- | --- | --- | --- | --- |
|  | | - **Categories** | - **Adjusted OR of death (95% CI)** | | - **P-value**^1^ |
|  |  |  |  |  |  |
| - **HL (lactate ≥5 mmol/L)** (stratum-specific effects of HL in each level of hyperglycaemia) | | | | | |
| - Hyperglycemia^2^ | - No | - HL, no | | - 1.00 (Ref.)^3^ | - <0.001 |
|  |  | - HL, yes | | - 8.55 (4.63, 15.78) |  |
|  | - Yes | - HL, no | | - 1.00 (Ref.)^3^ | - <0.01 |
|  |  | - HL, yes | | - 3.22 (1.56, 6.67) |  |
| - **Malaria** | | - No | | - 1.00 (Ref.)^3^ | - <0.001 |
|  |  | - Yes | | - 0.44 (0.28 0.67) |  |
| **Severe anaemia (Hb <5g/dL)** | | No | | - 1.00 (Ref.)^3^ | - 0.30 |
|  |  | Yes | | - 0.80 (0.50, 1.28) |  |
| **Level of consciousness** | | Alert | | - 1.00 (Ref.)^3^ |  |
|  |  | Prostration | | - 1.37 (0.70, 2.68) | - 0.40 |
|  |  | Coma | | - 6.72 (3.29, 13.72) | - <0.001 |
| - **Age (months)** | | - ≥ 12 | | - 1.00 (Ref.)^3^ | - <0.01 |
|  |  | - < 12 | | - 1.99 (1.23, 3.22) |  |
| - **Gender** | | - Male | | - 1.00 (Ref.)^3^ | - 0.90 |
|  |  | - Female | | - 1.02 (0.68, 1.52) |  |
| - **Site** | | - Mbale | | - 1.00 (Ref.)^3^ |  |
|  |  | - Kilifi | | - 1.52 (0.73, 3.13) | - 0.30 |
|  |  | - Mulago | | - 1.50 (0.82, 2.78) | - 0.20 |
|  |  | - Soroti | | - 1.14 (0.62, 2.10) | - 0.70 |
|  |  | - Lacor | | - - | - - |
|  |  | - Teule | | - 1.98 (0.85, 4.65) | - 0.10 |
| - **HIV test** | | - Negative | | - 1.00 (Ref.)^3^ | - 0.05 |
|  |  | - Positive | | - 2.16 (1.00, 4.65) |  |
| - **BUN (mmol/L)** | | - < 7.14 | | - 1.00 (Ref.)^3^ | - <0.001 |
|  |  | - ≥ 7.14 | | - 2.83 (1.78, 4.50) |  |
| - **Hyperglycaemia (glucose ≥8.3 mmol/L)** (stratum-specific effects of hyperglycaemia in each level of HL) | | | | | |
| - HL | - No | - Hyperglycaemia, no | | - 1.00 (Ref.)^3^ | - 0.16 |
|  |  | - Hyperglycaemia, yes | | - 1.69 (0.81, 3.52) |  |
|  | - Yes | - Hyperglycaemia, no | | - 1.00 (Ref.)^3^ | - 0.10 |
|  |  | - Hyperglycaemia, yes | | - 0.64 (0.38, 1.07) |  |

1. Chi-squared p-values
2. Hyperglycaemia was defined as a glucose level ≥ 8.3 mmol/L
3. Reference group

| **Multivariable analysis (logistic regression model) of clinical and laboratory factors on admission associated with death at 72 hours: fully adjusted OR (including interaction parameter between HL and Blood Urea Nitrogen (BUN))** | | | | |
| --- | --- | --- | --- | --- |
|  | | - **Categories** | - **Adjusted OR of death (95% CI)** | - **P-value**^1^ |
|  |  |  |  |  |
| - **HL (lactate ≥5 mmol/L)** (stratum-specific effects of HL in each level of BUN) | | | | |
| - BUN^2^ | - <7.14 | - HL, no | - 1.00 (Ref.)^3^ | - <0.001 |
|  |  | - HL, yes | - 9.57 (5.24, 17.50) |  |
|  | - ≥7.14 | - HL, no | - 1.00 (Ref.)^3^ | - 0.025 |
|  |  | - HL, yes | - 2.33 (1.11, 4.89) |  |
| - **Malaria** | | - No | - 1.00 (Ref.)^3^ | - <0.001 |
|  |  | - Yes | - 0.44 (0.28, 0.67) |  |
| **Severe anaemia (Hb <5g/dL)** | | No | - 1.00 (Ref.)^3^ | - 0.40 |
|  |  | Yes | - 0.82 (0.52, 1.30) |  |
| - **Level of consciousness** | | - Alert | - 1.00 (Ref.)^3^ |  |
|  |  | - Prostration | - 1.44 (0.74, 2.83) | - 0.3 |
|  |  | - Coma | - 6.87 (3.34, 14.14) | - <0.001 |
| - **Age (months)** | | - ≥ 12 | - 1.00 (Ref.)^3^ | - <0.01 |
|  |  | - < 12 | - 1.98 (1.22, 2.20) |  |
| - **Gender** | | - Male | - 1.00 (Ref.)^3^ | - 0.95 |
|  |  | - Female | - 1.01 (0.68, 1.51) |  |
| - **Site** | | - Mbale | - 1.00 (Ref.)^3^ |  |
|  |  | - Kilifi | - 1.54(0.74, 3.19) | - 0.25 |
|  |  | - Mulago | - 1.49 (0.81, 2.73) | - 0.20 |
|  |  | - Soroti | - 1.19 (0.65, 2.18) | - 0.58 |
|  |  | - Lacor | - - | - - |
|  |  | - Teule | - 1.94 (0.83, 4.54) | - 0.13 |
| - **HIV test** | | - Negative | - 1.00 (Ref.)^3^ | - 0.05 |
|  |  | - Positive | - 2.17 (1.01, 4.65) |  |
| - **Hyperglycaemia** | | No | - 1.00 (Ref.)^3^ | - 0.3 |
|  |  | Yes | - 0.78 (0.51, 1.21) |  |
| - **BUN** (stratum-specific effects of BUN in each level of HL) | | | | |
| - HL | - No | - BUN <7.14 | - 1.00 (Ref.)^3^ | - <0.001 |
|  |  | - BUN ≥7.14 | - 8.06 (3.76, 17.29) |  |
|  | - Yes | - BUN <7.14 | - 1.00 (Ref.)^3^ | - 0.01 |
|  |  | - BUN ≥7.14 | - 1.97 (1.17, 3.32) |  |

1. Chi-squared p-values
2. Elevated BUN was defined as a BUN level ≥ 7.14 mmol/L
3. Reference group

| **Multivariable analysis (logistic regression model) of clinical and laboratory factors on admission associated with death at 72 hours: fully adjusted OR (including interaction parameter between HL and crackles on auscultation)** | | | | | |
| --- | --- | --- | --- | --- | --- |
|  | | | - **Categories** | - **Adjusted OR of death (95% CI)** | - **P-value**^1^ |
|  |  |  |  |  |  |
| - **HL (lactate ≥5 mmol/L)** (stratum-specific effects of HL in each level of crackles) | | | | | |
| - Crackles on auscultation | | - No | - HL, no | - 1.00 (Ref.)^2^ | - <0.001 |
|  |  |  | - HL, yes | - 3.60 (2.09, 6.20) |  |
|  |  | - Yes | - HL, no | - 1.00 (Ref.)^2^ | - <0.001 |
|  |  |  | - HL, yes | - 29.45 (10.13, 85.55) |  |
| - **Malaria** | | | - No | - 1.00 (Ref.)^2^ | - 0.001 |
|  |  |  | - Yes | - 0.47 (0.31 0.73) |  |
| **Severe anaemia (Hb <5g/dL)** | | | No | - 1.00 (Ref.)^2^ | - 0.53 |
|  |  |  | Yes | - 0.86 (0.53, 1.39) |  |
| **Level of consciousness** | | | Alert | - 1.00 (Ref.)^2^ |  |
|  |  |  | Prostration | - 1.51 (0.76, 3.01) | - 0.24 |
|  |  |  | Coma | - 6.51 (3.13, 13.54) | - <0.001 |
| - **Hyperglycaemia** | | | - No | - 1.00 (Ref.)^2^ | - 0.51 |
|  |  |  | - Yes | - 0.86 (0.56, 1.34) |  |
| - **Age (months)** | | | - ≥ 12 | - 1.00 (Ref.)^2^ | - 0.02 |
|  |  |  | - < 12 | - 1.86 (1.12, 3.09) |  |
| - **Gender** | | | - Male | - 1.00 (Ref.)^2^ | - 0.86 |
|  |  |  | - Female | - 1.04 (0.69, 1.56) |  |
| - **Site** | | | - Mbale | - 1.00 (Ref.)^2^ |  |
|  |  |  | - Kilifi | - 1.81 (0.85, 3.83) | - 0.12 |
|  |  |  | - Mulago | - 1.63 (0.87, 3.07) | - 0.13 |
|  |  |  | - Soroti | - 1.35 (0.72, 2.55) | - 0.35 |
|  |  |  | - Lacor | - - | - - |
|  |  |  | - Teule | - 2.48 (1.03, 5.93) | - 0.04 |
| - **HIV test** | | | - Negative | - 1.00 (Ref.)^2^ | - 0.03 |
|  |  |  | - Positive | - 2.31 (1.07, 5.00) |  |
| - **BUN (mmol/L)** | | | - < 7.14 | - 1.00 (Ref.)^2^ | - <0.001 |
|  |  |  | - ≥ 7.14 | - 3.08 (1.93, 4.92) |  |
| - **Crackles on auscultation** (stratum-specific effects of crackles in each level of HL) | | | | | |
| - HL | - No | | - Crackles, no | - 1.00 (Ref.)^2^ | - 0.10 |
|  |  |  | - Crackles, yes | - 0.44 (0.16, 1.18) |  |
|  | - Yes | | - Crackles, no | - 1.00 (Ref.)^2^ | - <0.001 |
|  |  |  | - Crackles, yes | - 3.57 (1.97, 6.46) |  |

1. Chi-squared p-values
2. Reference group
