# Supplementary figures and images for: Lactate clearance as a prognostic marker of mortality in severely ill febrile children in East Africa
Source: BMC Med. 2018 Mar 9;16:37. doi: 10.1186/s12916-018-1014-x (PMC5844084; doi:10.1186/s12916-018-1014-x)

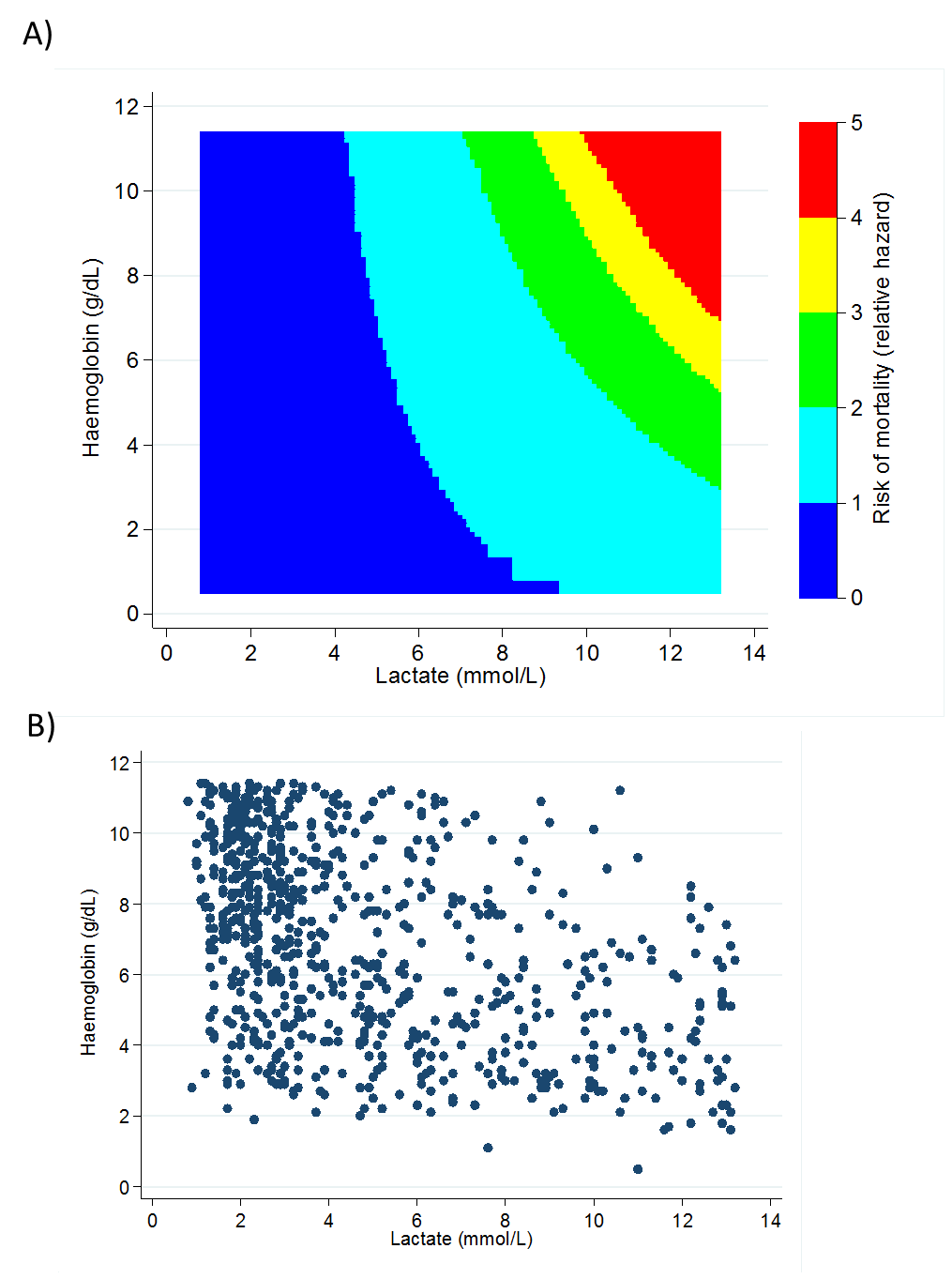

Supplement: Supplementary file 2 — Plots of the relationship between haemoglobin and lactate and mortality estimated from the adjusted Cox regression model. Originally published in BMC Medicine 13:174 by George et al. [5]. (PNG 80 kb) [file 12916_2018_1014_MOESM2_ESM.png]
